# Two new species of *Tasiocera* Skuse from Baltic amber (Diptera, Limoniidae)

**DOI:** 10.1038/s41598-023-51033-z

**Published:** 2024-01-03

**Authors:** Iwona Kania-Kłosok, Patryk Wąsacz, Michał Tuchowski, Wiesław Krzemiński

**Affiliations:** 1 Institute of Biology, University of Rzeszów, Rzeszów, Poland; 2 Institute of Systematics and Evolution of Animals, Polish Academy of Sciences, Kraków, Poland

**Keywords:** Evolution, Environmental sciences

## Abstract

The paper presents very rare fossil record of crane flies. Inclusions in Baltic amber have documented the second evidence of the existence of the genus *Tasiocera* (Diptera: Limoniidae: Chioneinae) in ancient epochs of Earth’s history. Hypotheses were also introduced regarding the environmental preferences of fossil *Dasymolopilus* in relation to modern representatives of the subgenus. The genus is numerous in species and widespread in the modern fauna, while only one species, *Tasiocera* (*Dasymolophilus*) *circumcincta*, has been known from the fossil record to date. The paper presents an additional description and graphic documentation of this species based on new materials. The discovery of two other species *Tasiocera* (*Dasymolophilus*) *gorskii* sp. nov. and *Tasiocera* (*Dasymolophilus*) *baltica* sp. nov. in Eocene resins documents the occurence of *Tasiocera* since Eocene. Interestingly, only subgenus *Dasymolophilus* is represented both in extinct and extant fauna while subgenus *Tasiocera* is not known from the fossil record. The new discovery is very significant, and the result of the study adds a brick to our understanding of the evolution of this group of insects and their habitat.

## Introduction

Subfamily Chioneinae Rondani^1^ (= Eriopterinae van der Wulp^2^; see Starý^3^) is represented in recent fauna by 60 genera, 81 subgenera and over 4400 species^4^. It is the largest subfamily of Limoniidae in respect of the numer of species. The oldest representative of Chioneinae is *Gonomyia* (*Azaria*) *libanensis* Kania, Krzemiński and Krzemińska^5^. The holotype of this species was found in Hammana–Mdeyrij outcrop, Caza (District) Baabda, Mouhafazet Jabal Loubnan (Governorate Mount Lebanon), Central Lebanon and it is dated on Early Cretaceous, ante-Barremian^6^. From Cretaceous period are known also the other Chioneinae, the representatives of *Rhabdomastix* Skuse^7^, subgenus *Myanmamastix* Kania-Kłosok, Jordan-Stasiło, Kopeć, Janiszewska & Krzemiński^8^*,* preserved in Myanmar amber dated on Upper Cretaceous ca. 98.79 ± 0.62 Ma. Most of the Chioneinae known from the fossil record have been preserved in Eocene Baltic amber, such as representatives of *Cheilotrichia* Rossi^9^ or *Ormosia* Rondani^10^. Few species are known from the younger periods of the Oligocene of Germany, such as *Erioptera* Meigen^11^ or from the Miocene, such as *Styringomyia* Loew^12^ (Miocene Italy) or *Molophilus* Curtis^13^ (Dominican amber).

The genus *Tasiocera* Skuse^7^ is very rare in fossil record. Only one species belonging to the genus has been described so far, *Tasiocera* (*Dasymolophilus*) *circumcincta* Meunier^14^. The species abundance of the modern fauna of the genus does not correspond to that known from the fossil record. In recent fauna two subgenera are represented within the genus *Tasiocera*—*Tasiocera* Skuse^7^ (with 40 recent species) and *Dasymolophilus* Goetghebuer^15^ (35 recent species). The representatives of the subgenus *Tasiocera* are found only in Australia and New Zealand. In the case of the subgenus *Dasymolophilus* a wider range is noted, with a large number of species occuring in the Ethiopian and Palearctic zoogeographical regions^4^ (Table 1).

Crane flies belonging to the subgenus *Dasymolophilus* are characterized by elongated flagellomeres of antennae, the wings are rather narrow, covered with long and dense setae, both on the wing edges and along the internal veins. The cubital and medial veins are costalized in the distal part of the wing and are divided, the anal vein A_2_ is shortened, and the discal cell (d-cell) is usually open. The gonostyles are often elongate, and the aedeagus complex can vary greatly between species, but is often asymmetric^17^ and the postnotum bristles are present in the representatives of the *Dasymolophilus* group^18^.

The classification of the subgenus *Dasymolophilus* has changed, the subgenus *Dasymolophilus* was originally included in the genus *Molophilus.* Alexander^19^ and Edwards^20^ showed a closer relationship of this taxon to the genus *Tasiocera* based on differences in morphology. Characteristics such as the different structure of gonostylus than in *Molophilus*, and the presence of postnotum bristles in the representatives of the subgenus *Dasymolophilus* turned out to be decisive here. In 1994 Evenhuis^21^ classified *Dasymolophilus* as a subgenus of the genus *Tasiocera* alongside the subgenus *Tasiocera*. Currently, this group of flies represents the genus *Tasiocera.*

**Table 1:** A table of biogeographical distribution of subgenus *Dasymolophilus* (after Oosterbroek^4^, ** after Starý, in Jedlička et al.^22^).

Area of occurrance	Number of species for individual countries
*Dasymolophilus*/35 recent species	
AL, AZ, BE, BR, CA, CG, CL, EE, GR, JP, KE, KM, KP, KZ, MA, MY, NL, PT, RE, RS, RW, SC, TR, TZ, UA*	1
AU, DK, ES, LT, MK, NO, SE	2
BG, CH, CN, FI, IE, PL, RO, RU	3
FR, HU, MG, UG	4
CZ, DE, GB, SK**, US, ZA	5
IT	6
*Tasiocera*/40 recent species	
PG	1
ID	2
NZ	12
AU	25

*Alpha code—2 country names used according to the ISO 3166-1 standard of the International Organization for Standarization ISO (1997).

So far, on the basis of three specimens, two males and female, from the historical Klebs collection preserved as inclusions in Eocene Baltic amber (Table 2), one species has been described from fossil record. This species was initially classified into the genus *Erioptera*, subgenus *Haplolabis* and described as *Erioptera* (*Haplolabis*) *circumcincta* Meunier^14^, however it was later transferred to the genus *Tasiocera*, subgenus *Dasymolophilus*.

**Table 2:** List of the fossil specimens of the genus *Tasiocera* studied so far.

Species	Specimen number	Sex	Type of material	Age/origin	Collection
*Tasiocera* (*Dasymolophilus*) *circumcincta* Meunier^14^	3934.26 (lectotype)	♂	inclusion	Eocene/Baltic amber	Klebs coll
	1850	♀	inclusion	Eocene/Baltic amber	Klebs coll
	3967	♂	inclusion	Eocene/Baltic amber	Klebs coll

New data enrich our knowledge about evolutionary history of *Dasymolophilus*. It is not without significance that the genus is very rare in the fossil record. All the more valuable is any information about it found in the fossil resins. The paper present the second evidence of *Dasymolophilus* in the ancient ages of Earth’s history.

## Material and methods

Research material were four inclusions in Baltic amber (three males and one female) housed in the collection of Institute of Systematics and Evolution of Animals, Polish Academy of Sciences, Kraków, Poland (ISEA PAS).

The specimens were studied using Nikon SMZ 1500 and a camera Nikon DS-Fi1. The measurements were taken with NIS-Elements D 3.0 only for undamaged body parts. Such measurments as d-cell length were given from its posterior edge to the point of connection of vein m-m with vein M_3_. Drawings were completed based on the specimen and photographs by I.K.-K. The wing venation nomenclature follows that of Krzemiński and Krzemińska^23^, the terminology for the structures of the hypopygium is in accordance with McAlpine et al.^24^.

### Nomenclatural acts

The electronic edition of this article conforms to the requirements of the amended International Code of Zoological Nomenclature, and hence the new names contained herein are available under that Code from the electronic edition of this article. This published work and the nomenclatural acts it contains have been registered in ZooBank, the online registration system for the ICZN. The ZooBank LSIDs (Life Science Identifiers) can be resolved and the associated information viewed through any standard web browser by appending the LSID to the prefix ‘http://zoobank.org/’. The LSID for this publication is: urn:lsid:zoobank.org:pub:851A23EE-5D98-4556-AD8F-E01288A95F3F.

## Results

Systematic palaeontology

Order Diptera Linnaeus^25^.

Infraorder Tipulomorpha Rohdendorf^26^.

Family Limoniidae Speiser^27^.

Subfamily Chioneinae Rondani^28^.

Genus *Tasiocera* Skuse^7^.

Type species: *Tasiocera tenuicornis* Skuse^7^ (designation: Alexander^29^).

Subgenus *Dasymolophilus* Goetghebuer^15^.

Type species: *Erioptera murina* Meigen^30^ (original designation).

### Tasiocera (Dasymolophilus) gorskii sp. nov.

**Figure 1:**
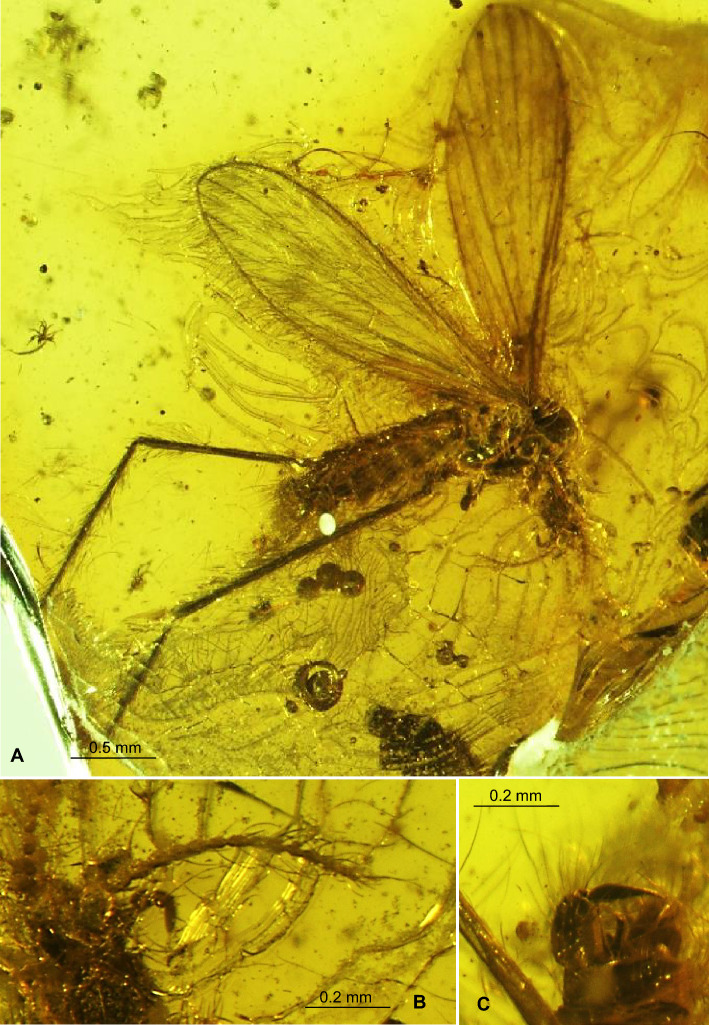
*Tasiocera* (*Dasymolophilus*) *gorskii* sp. nov., No. MP/7709 (male) holotype (coll. A. Górski; ISEA PAS): (**A**) body, lateral view; (**B**) antenna and palpus; (**C**) gonocoxite and gonostylus.

**Figure 2:**
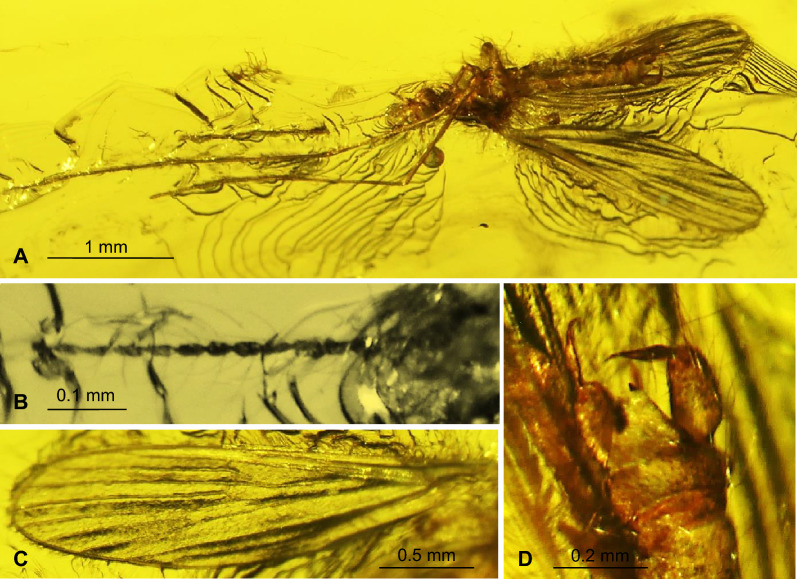
*Tasiocera* (*Dasymolophilus*) *gorskii* sp. nov., No. MP/3438 (male) paratype (ISEA PAS): (**A**) body, ventral view; (**B**) antenna; (**C**) wing; (**D**) hypopygium.

**Figure 3:**
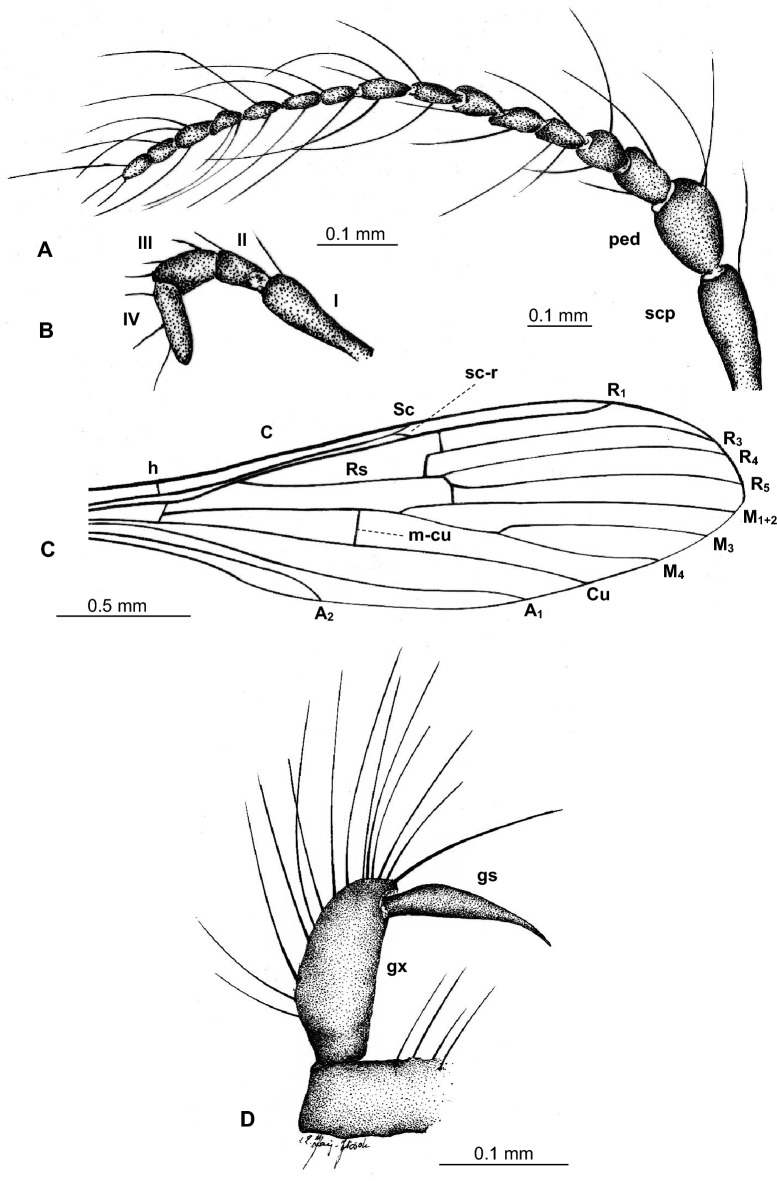
*Tasiocera* (*Dasymolophilus*) *gorskii* sp. nov., No. MP/7709 (male) holotype (coll. A. Górski; ISEA PAS): (**A**) antenna; (**B**) palpus; (**C**) wing; (**D**) gonocoxite and gonostylus. *scp* scape, *ped* pedicel, *I–IV* palpomeres 1–4, *gx* gonocoxite, *gs* gonostylus.

**Figure 4:**
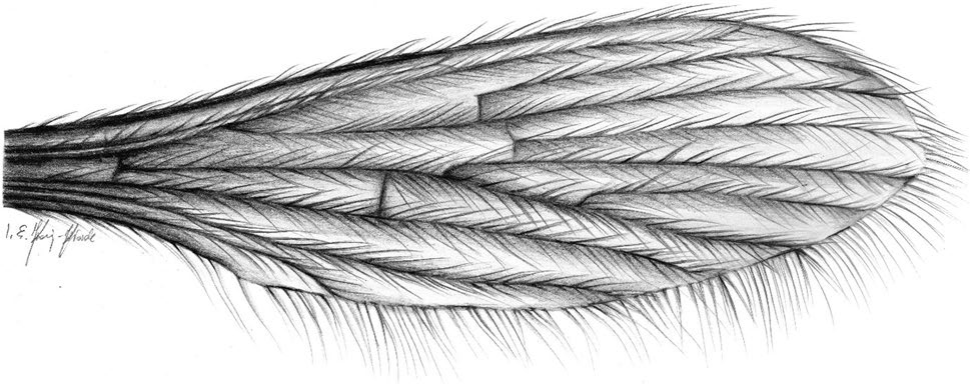
*Tasiocera* (*Dasymolophilus*) *gorskii* sp. nov., No. MP/7709 (male) holotype (coll. A. Górski; ISEA PAS); wing, reconstruction.

(Figs. 1, 2, 3, 4) LSID urn:lsid:zoobank.org:act:6CCDB888-3437-477E-84A8-39631F538107.

#### Diagnosis

Wing without spots; tip of Sc just before fork of Rs; sc-r in one of its length from edge of wing; Rs arrange more than half of Sc; r-r (R_2_) situated beyond fork of Rs; basal section of R_5_ (from fork of R_4+5_ to connection of r-m) shorter than r-m; R_1_ reaching the edge of wing before tip of M_4_; crossvein m-cu situated before fork of Mb, at a distance equal to its length, beyond tip of A_2_.

#### Etymology

The specific name is dedicated to Andrzej Górski (Bielsko Biała, Poland) the owner of huge collection of inclusions and the specialist on fossil and recent entomofauna.

#### Material examined

Holotype No. 7709 (male), col. Andrzej Górski (ISEA PAS); paratype No. MP/3438 (ISEA PAS).

#### Horizon and locality

The age range of Baltic amber is still debatable, but the most current state of knowledge is that it is of Priabonian age, between 38 and 34 million years (based on pollen, spores and phytoplankton of the amber embedding layer, the so-called Blue Earth)^31^.

#### Description

Body length 2.75 mm (holotype), 2.50 mm (paratype), brown.

Head: 0.28 mm wide (holotype), 0.24 mm wide (paratype); antenna (Figs. 1, 2, 3) 0.88 mm long (1/0.08; 2/0.10; 3/0.07; 4/0.05; 5/0.05; 6/0.05; 7/0.05; 8/0.05; 9/0.05; 10/0.05; 11/0.05; 12/0.05; 13/0.05; 14/0.05; 15/0.05; 16/0.05) (holotype), 0.67 mm long (paratype), with scape elongate, narrow, only slightly widened distaly, pedicel not very elongate, barrel-like, 1.5× longer than its wide; scape and pedicel with a few elongate setae longer than segments bearing them; first flagellomere massive, rather cylindrical, wider than other, flagellomeres slightly tapered at the base, became more slender to the apex of antenna, last flagellomere shorter than penultimate one; setae on flagellomeres very elongate, even 6× longer than segments bearing them. Palpus (Figs. 1, 2, 3) 0.29 mm long (holotype), 0.26 mm long (paratype), last palpomere elongate, only slightly longer than penultimate one, palpomeres 1–3 slightly tapered at the base.

Thorax: wing (Figs. 1, 2, 3, 4) 2.43 mm long, 0.71 mm wide (holotype), 2.84 mm long, 0.68 mm wide (paratype), length of wing approximately 3× of its width; tip of Sc approximately in half the length of wing, before crossvein r-m; vein Mb longer than M_3_; distal part of Cu from the point of connection of Cu with crossvein m-cu to the edge of wing almost straight; tip of A_2_ distincly before half the length if wing, in 0.3× the length of Rs from fork of Rb. Haltere 0.35 mm long (holotype), 0.36 mm long (paratype).

Abdomen: hypopygium (Figs. 1, 2, 3) 0.31 mm long; gonocoxite 0.16 mm long; gonostylus 0.15 mm long (holotype); 0.36 mm long; gonocoxite 0.21 mm long; gonostylus 0.15 mm long (paratype). Gonostylus slightly longer than gonocoxite, pointed at apex, widened in midlength; gonocoxite 2× as long as wide.

**Figure 5:**
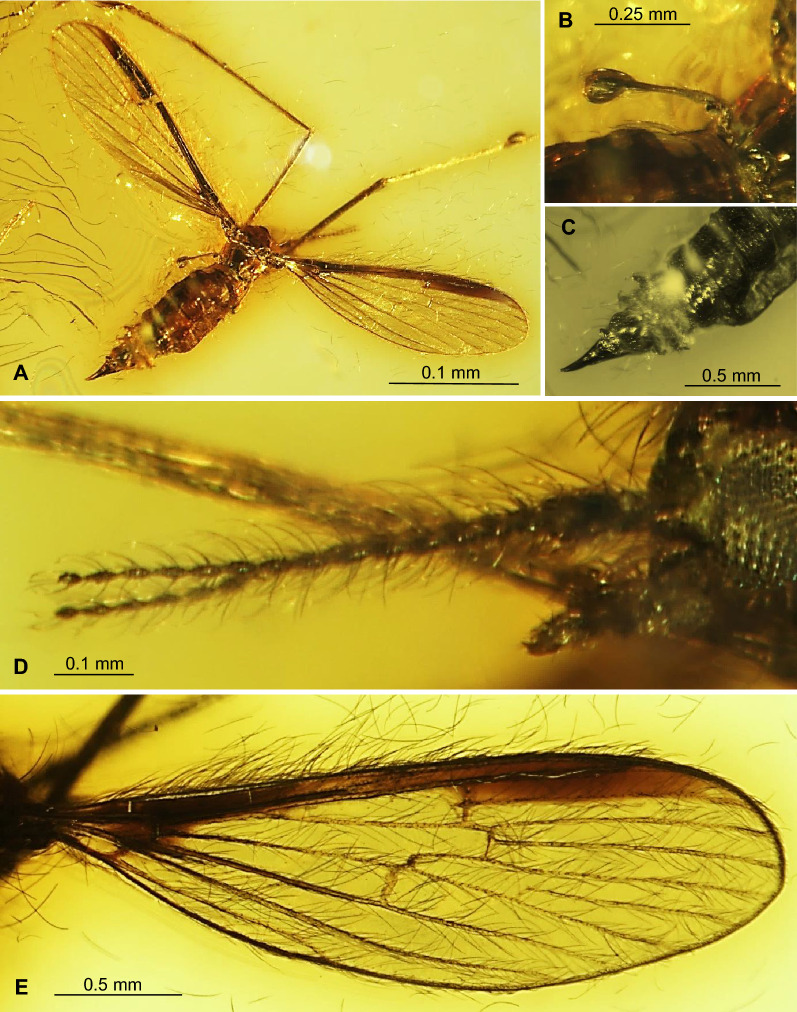
*Tasiocera* (*Dasymolophilus*) *baltica* sp. nov., No. MP/3106 (female) holotype (ISEA PAS); (**A**) body, dorsal view; (**B**) haltere; (**C**) ovipositor; (**D**) antenna; E. wing.

**Figure 6:**
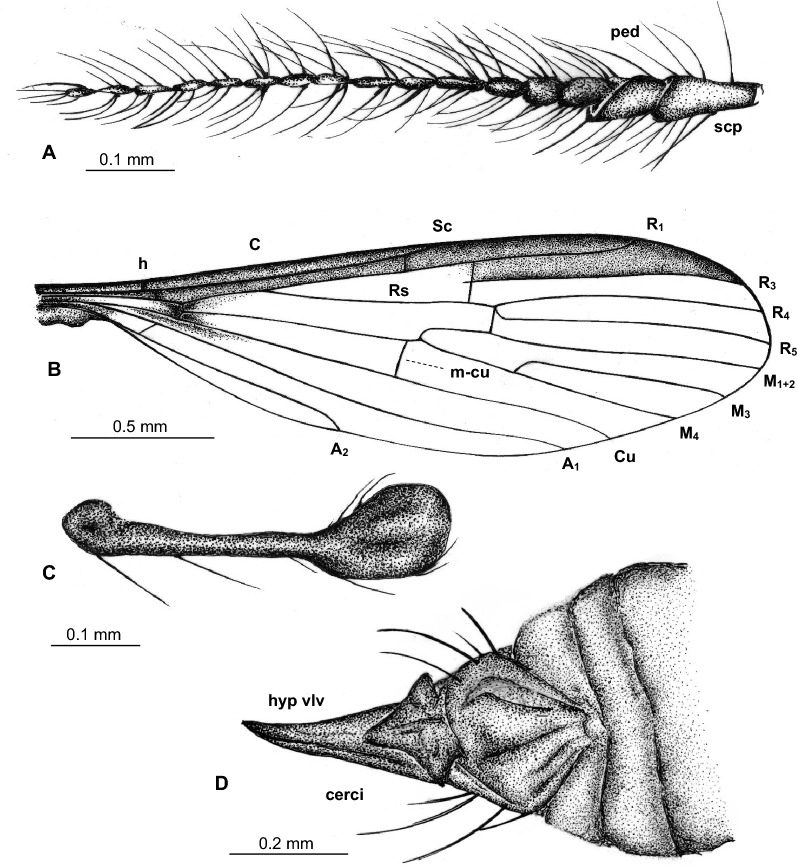
*Tasiocera* (*Dasymolophilus*) *baltica* sp. nov., No. MP/3106 (female) holotype (ISEA PAS); (**A**) antenna; (**B**) wing; (**C**) haltere; (**D**) ovipositor.

*Comparison.* In *T.* (*D.*) *gorskii* sp. nov. and in *T.* (*D.*) *circumcincta* wings are pale without color pattern (see Figs. 1, 2, 3, 4) while in *T.* (*D.*) *baltica* sp. nov. occur distinct wide dark colored stripe along costal vein (C) (see Figs. 5, 6). There are also some important differences in wing venation, the tip of Sc *T.* (*D.*) *gorskii* sp. nov. is positioned before fork of Rs while in *T.* (*D.*) *circumcincta* tip of Sc is positioned beyond fork of Rs, in approximately 0.14× the length of R_1_. Crossvein sc-r in *T.* (*D.*) *gorskii* sp. nov. is situated one of its length from edge of wing, in *T.* (*D.*) *baltica* sp. nov. this vein occur in two of its length from edge of wing. In *T.* (*D.*) *gorskii* sp. nov. and *T.* (*D.*) *baltica* sp. nov. Rs arrange more than half of Sc, in *T.* (*D.*) *circumcintca* Rs is not longer than half of Sc. In (*D.*) *gorskii* sp. nov. r-r (R_2_) is situated beyond fork of Rs while in *T.* (*D.*) *baltica* sp. nov. and *T.* (*D.*) *circumcincta* r-r (R_2_) is situated at the same level as fork of Rs, basal section of R_5_ is shorter than r-m in *T.* (*D.*) *baltica* sp. nov. and (*D.*) *gorskii* sp. nov. and equal in length in *T.* (*D.*) *circumcincta*. Moreover, in *T.* (*D.*) *gorskii* sp. nov. and *T.* (*D.*) *baltica* sp. nov. R_1_ reaching the edge of wing before tip of M_4_, in *T.* (*D.*) *circumcincta* beyond. In *T.* (*D.*) *gorskii* sp. nov. and *T.* (*D.*) *baltica* sp. nov. crossvein m-cu is positioned before fork of Mb, in *T.* (*D.*) *circumcincta* at this fork or just before.

### Tasiocera (*Dasymolophilus*)* baltica* sp. nov.

(Figs. 5, 6) LSID urn:lsid:zoobank.org:act:1EDE363F-5C99-41F3-A11C-97D543494FAC.

#### Diagnosis

Wing with distinct wide trail along costal vein (C); tip of Sc before fork of Rs, in approximately 0.2× the length of Rs from its fork, sc-r in two of its length from edge of wing; Rs arrange more than half of Sc; r-r (R_2_) situated at fork of Rs; basal section of R_5_ (from fork of R_4+5_ to connection of r-m) shorter than r-m; tip of R_1_ reaching the edge of wing before tip of M_4_; crossvein m-cu before fork of Mb, at a distance shorter than its length, beyond tip of A_2_.

#### Remarks

Due to the morphology of the wing venation, it became possible to identify a new species based on the characteristics of the female. The newly designated species differs significantly in wing venation from the already known species *Tasiocera* (*Dasymolophilus*) *circumcincta* Meunier^14^ and also newly described herein species *T.* (*D.*) *gorskii* sp. nov. Alexander^32^ gives the characteristics of the venation of both males and females, so it was possible to compare the venation of the wings, taking into account sexual dimorphism in some species of Diptera.

#### Etymology

The specific name ‘*baltica*’ is derived from Baltic amber.

#### Material examined

Holotype: No. MP/3106 (female), haused in the Institute of Systematics and Evolution of Animals, Polish Academy of Sciences, Kraków (ISEA PAS).

#### Horizon and locality

The age range of Baltic amber is still debatable, but the most current state of knowledge is that it is of Priabonian age, between 38 and 34 million years (based on pollen, spores and phytoplankton of the amber embedding layer, the so-called Blue Earth)^31^.

#### Description

Body length 2.75 mm, brown, pterostigma present.

Head: 0.20 mm wide; antenna (Figs. 5, 6) 0.88 mm long (1/0.08; 2/0.10; 3/0.07; 4/0.05; 5/0.05; 6/0.05; 7/0.05; 8/0.05; 9/0.05; 10/0.05; 11/0.05; 12/0.05; 13/0.05; 14/0.05; 15/0.05; 16/0.05) with scape elongate, narrow at base, widened distaly, and not very elongate, cylindrical pedicel, 1.5× longer than its wide; scape and pedicel with a few elongate setae, setae on pedicel longer than seagment bearing them; first flagellomere massive, wider than other, flagellomeres 2–13 approximately the same length, last flagellomere shorter than penultimate; setae on flagellomeres 1–14 longer than segments bearing them. Palpus (Figs. 5, 6) 0.25 mm long, rather short, all segment almost equal in length.

Thorax: wing (Figs. 5, 6) 2.70 mm long, 0.82 mm wide, length of wing 3.5× of its width; crossvein (h) situated in 0.16× the length of wing; crossvein sc-r situated in 2× of its length from the tip of Sc; tip of Sc before half the length of wing, before crossvein r-m and before fork of Rs; fork of Rb approximately in 0.3× the length of Mb; vein Mb approximately as long as M_3_; distal part of Cu from the point of connection of Cu with crossvein m-cu to the edge of wing curved; tip of A_2_ distincly before half the length if wing, in 0.3× the length of Rs from fork of Rb. Haltere 0.43 mm long.

Abdomen: ovipositor (Figs. 5, 6) 0.52 mm long; hypogynal valvae and cerci only slightly elongate, rather wide, comparable length.

#### Comparison

In *T.* (*D.*) *circumcincta* Meunier^14^ and *T.* (*D.*) *gorskii* sp. nov., pterostigma is absent, the color pattern also does not occur, countrary to *T.* (*D.*) *baltica* sp. nov., where well visible, darkbrown streak occur along to costal vein, pterostigma is presented. There are also many differences in wing venation, see ‘Comparison’ under *T.* (*D.*) *gorskii* sp. nov.

### *Tasiocera *(*Dasymolophilus*)* circumcincta* Meunier^14^

1906. *Erioptera* (*Hoplolabis*) *circumcincta* Meunier^14^: 368–369

1931. *Dasymolophilus circumcinctus* Meunier^14^: Alexander^32^: 98

1994. *Tasiocera* (*Dasymolophilus*) *circumcincta* Meunier^14^: Evenhuis^21^: 34

(Figs. 7, 8).

**Figure 7:**
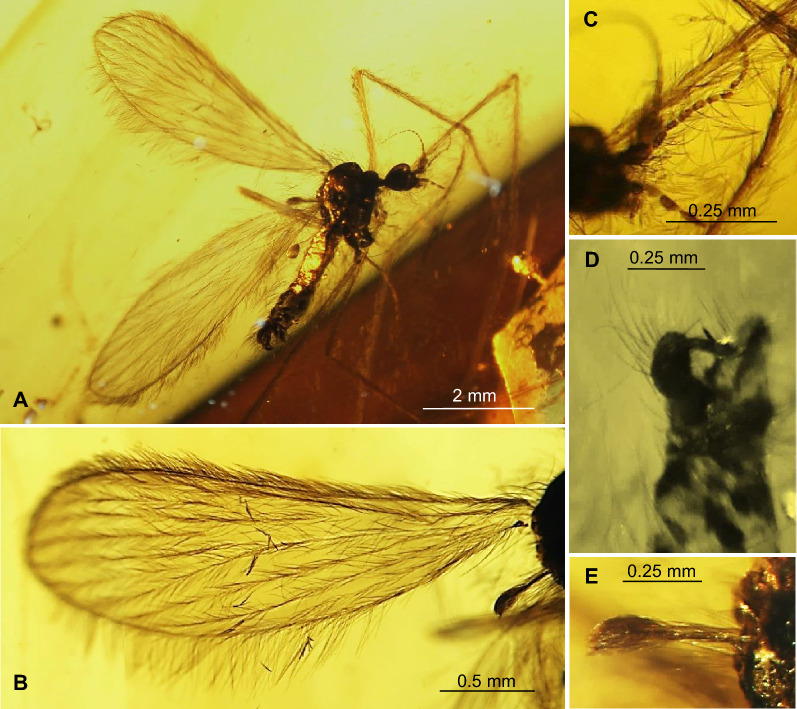
*Tasiocera* (*Dasymolophilus*) *circumcincta* Meunier, 1906, No. MP/3085 (male), additional material (ISEA PAS): (**A**) body, lateral view; (**B**)wing; (**C**) antenna and palpus; (**D**) hypopygium; (**E**) haltere.

**Figure 8:**
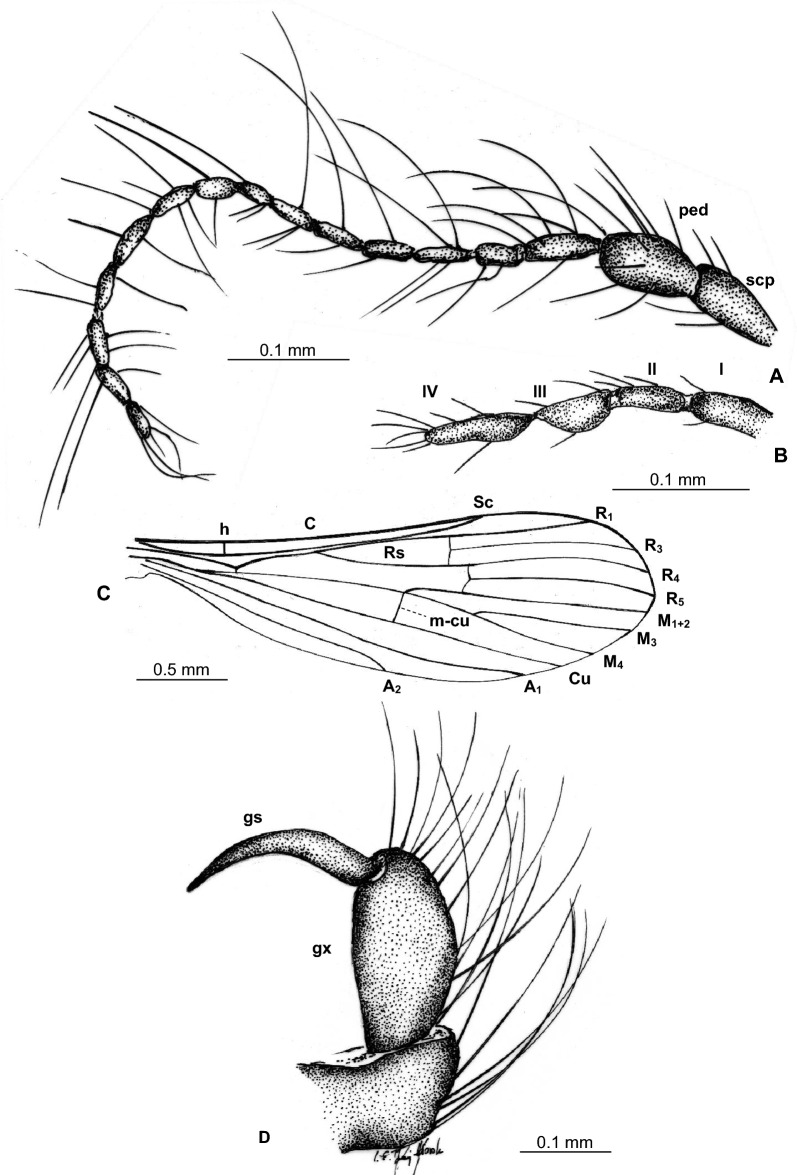
*Tasiocera* (*Dasymolophilus*) *circumcincta* Meunier, 1906, No. MP/3085 (male), additional material (ISEA PAS): (**A**) antenna; (**B**) palpus; (**C**) wing; (**D**) gonocoxite and gonostylus. *scp* scape, *ped* pedicel, *I–IV* palpomeres 1–4, *gx* gonocoxite, *gs* gonostylus.

#### Diagnostic characters

Wing without coloration; tip of Sc beyond fork of Rs, in approximately 0.14× the length of R_1_; Rs not longer than half of Sc; r-r (R_2_) situated at fork of Rs; basal section of R_5_ (from fork of R_4+5_ to connection of r-m) equal in length to r-m; tip of R_1_ reaching the edge of wing beyond tip of M_4_; crossvein m-cu at fork or just before fork of Mb, before tip of A_2_.

#### Material examined

No. MP/3085 (male), deposited in the Institute of Systematics and Evolution of Animals, Polish Academy of Sciences, Kraków (ISEA PAS).

#### Horizon and locality

The age range of Baltic amber is still debatable, but the most current state of knowledge is that it is of Priabonian age, between 38 and 34 million years (based on pollen, spores and phytoplankton of the amber embedding layer, the so-called Blue Earth)^31^.

#### Additional description

Body 2.5 long, brown, wings without spots, pterostigma absent.

Head: width of head 0.27 mm; antenna (Figs. 7, 8) 0.77 mm long (1/0.07; 2/0.10; 3/0.06; 4/0.05; 5/0.05; 6/0.04; 7/0.04; 8/0.04; 9/0.04; 10/0.04; 11/0.04; 12/0.04; 13/0.04; 14/0.04; 15/0.04; 16/0.04) with elongate scape, tapered at base, slightly wider distally; not numerous, scape with slightly elongate setae, but shorter than segment bearing them, pedicel with a few elongate setae, two of them much longer than segment bearing them; on all flagellomeres a few very elongate seta, much longer than the length of segments bearing them. Palpus (Figs. 7, 8) 0.24 mm long; palpomeres 1–2 narow, slightly elongate, third palpomeres shorter than the last one, widened in midlength, last palpomeres slightly elongate, 4× as long as wide. On palpomeres a few slightly elongate setae, shorter than segments bearing them.

Thorax: wing (Figs. 7, 8) 2.65 mm long, 0.85 mm wide, the length of the wing corresponds to 3.5× its width, crossvein (h) situated at a distance corresponding to approximately 1/5 of the length of the wing, measured from its base; tip of vein Sc beyond half the length of wing, opposite crossvein r-m, beyond fork of Rs; fork of Rb approximately in half length of Mb; Mb longer than M_3_; distal part of Cu from the point of connection of Cu with crossvein m-cu to the edge of wing almost straight; tip of A_2_ in approximately in half the length of wing, just beyond half the length of Rs. Haltere short, the length of haltere 0.51 mm.

Abdomen: hypopygium (Figs. 7, 8) 0.38 mm long, gonocoxite narrow, elongate, 0.19 mm long, gonostylus 0.09 mm long, narrow, elongate, tipped.

### Key to species of *Dasymolophilus* known from fossil record in respect to subgenera

1. No macrotrichia on the wing membrana ***Tasiocera***
  The presence of macrotrichia on the wing membrana … *Dasymolophilus* (2)
2. Tip of Sc before fork of Rs; Rs arrange more than half of Sc; basal section of R_5_ (from fork of R_4+5_ to connection of r-m) shorter than r-m; R_1_ reaching the edge of before tip of M_4_; crossvein m-cu far before fork of Mb … (3)
  Tip of Sc beyond fork of Rs; Rs shorter than half the lenght of the vein Sc; basal section of R_5_ (from fork of R_4+5_ to connection of r-m) equal to r-m; R_1_ reaching the edge of beyond tip of M_4_; crossvein m-cu at or just before fork of Mb … *T.*(*D.*) *circumcincta* (Figs. 7, 8).
3. Wing with distinct wide dark colored stripe along costal vein (C); sc-r in two of its length from edge of wing; r-r (R_2_) at fork of Rs … *T.* (*D.*) *baltica* sp. nov. (Figs. 5, 6).
  Wing without coloration; sc-r in one of its length from edge of wing; r-r (R_2_) beyond fork of Rs … *T.* (*D.*) *gorskii* sp. nov. (Figs. 1–4).

## Discussion

The fossil record often provides detailed information about the morphology of extinct organisms, the history of their differentiation and shows direct evidence of evolutionary changes in many groups, also about the environment in the past epochs of Earth's history^16^. The most numerous group of animals in fossil resins of various ages are insects, among them representatives of Diptera. The fossil record proves that they were numerous starting from the Triassic^23,33^. Many extinct Limoniidae are known from the fossil record, and in the modern fauna there are such taxa of craneflies that the oldest representatives appeared not so early, for example, in the Eocene, like *Elephantomyia* Osten Sacken^34^, but also those known since the older periods like Cretaceous *Helius*^35^. Some genera of Limoniidae are very rare in the fossil record. The genus *Tasiocera* is represented in the modern fauna by over 70 species, but only one species has been known from the fossil record so far. The more valuable are the discoveries of new species for science, which is not without significance, for example, when reconstructing the evolutionary paths of the group of insects they represent, but also drawing conclusions about the environment in which these insects lived, including climatic conditions. In the Eocene, the northern part of today’s Europe, about 40 Ma, was covered with mixed forests. In addition to trees of the genus *Pinus*, which probably produced large amounts of resin and were characteristic of a temperate or rather cold climate, there were sequoias and flowering plants typical of a hot climate. There are many examples confirming that in the Middle Eocene, about 30–40 Ma (this is more or less, how the age of Baltic amber is defined) insects preferring tropical and subtropical climates existed in the north of Europe^16^.

*Tasiocera* crane flies are present on all continents except Antarctica, but they are most numerous in Australia and Oceania and in the Ethiopian zoogeographical region (Table 1), which may explain their rare occurrence in Baltic amber. The oldest representatives of the genus *Tasiocera* are known from Baltic amber, so the beginning of the evolution of this group can be traced back to the Eocene period. In the Cretaceous some Limoniidae underwent rapid radiation with the adaptation to the new food spectrum. Flowering plants (Angiospermae) began to spread in this period, flies, belonging to the genus *Helius*, the oldest representatives of the subfamily Limoniinae (family Limoniidae, Nematocera), already at that time developed elongated mouthparts used to feed most likely on the nectar of flowers. The oldest species of the genus *Helius* are known from the Early Cretaceous Lebanese amber, such as *Helius ewa* Krzemiński, Kania & Azar^36^ with a characteristic elongated rostrum^36^.

However, within the Limoniidae there are also known taxa that appeared much later, in the Eocene, as *Tasiocera* represented in fossil record only by the subgenus *Dasymolophilus*.

In the modern fauna out of the two subgenera distinguished within the genus *Tasiocera*—*Tasiocera* and *Dasymolophilus* much more biogeographically distributed is the subgenus *Dasymolophilus*. Slightly more diverse in terms of numer of species, but occurring in a relatively much smaller area (Australia and Oceania), is the subgenus *Tasiocera*, unrepresented in the fossil record. So far, there are no fossil records of flies of the genus *Tasiocera*, subgenus *Tasiocera*. The occurence of representatives of the *Tasiocera* subgenus only in Australia and Oceania may probably be due to the long geological isolation of the Austalian continent. Australia as a continent was part of Gondwana during the Jurassic period. The separation of India, Africa and Madagascar from this supercontinent meant that at one point it was only Antarctica, Australia and South America. The separation of Australia from the other two continents occurred about 96 Ma. As long as Australia was connected to or near Antarctica, warm air masses from Australia moved towards the South Pole. The Australian climate at that time was warm and humid. However, the northward drift of Australia around 46 Ma (thus increasing the distance between Australia and Antarctica) made the climate much drier. This proces was most intensified when Australia moved into tropical zone. Since the separation of Australia from Antarctica, its geological isolation began resulting in the emergence of a characteristic fauna on the Australian continent with a significant proportion of endemics^37^.

One previously known species *T.* (*D.*) *circumcincta* and two other described herein representing the genus *Tasiocera* were described on the basis of inclusions in Eocene Baltic amber, but the demonstration of the presence of antoher species of the subgenus *Dasymolophilus* in the former tropical Eocene forests of Europe shows that an even greater species diversity of this subgenus was possible in ancient epochs of Earth's history. The reasons for the absence of representatives of the *Tasiocera* subgenus in Baltic amber are probably to be sought in the presence of a significant geographical barier in the past.

Finding a new species is very significant, it allows for a better understanding of the group, evolutionary paths or phylogenetic relationships. Although one of the newly described species determined on the basis of the female morphological features, such as different wing venation or the presence of dark brown pattern of wing, leave no doubt that it is a representative of a different species than known from the fossil record so far.

Amber gives us the opportunity to see organisms that lived in the past. The bodies of insects preserved as an inclusions in different age of amber are three-dimensional preparations created by nature itself. Even if the original colors have not been preserved in amber, it is possible to determine the origin of color spots, for example on wings, and distinguish them from artifacts that sometimes occur in amber. An understanding of the taphonomic factors that control the preservation of color is key to assessing the fidelity with which original colors are preserved and can constrain interpretations of the visual appearance of fossil insects. Experimental taphonomic studies inform on how color alters during diagenesis. Preservation of color is controlled by a suite of factors, the most important of which relate to the diagenetic history of the host sediment, also color preservation relating to cuticular pigments in insects^38^.

The color of the stripes on the wings of *T.* (*D.*) *baltica* sp. nov. probably changed durring the fossilization process, but the shape of the colored area has been preserved. It is not artifact, even if the wings are not colored in modern members of the genus *Tasiocera*. The species *T.* (*D.*) *baltica* sp. nov. is easily distinguishable from other members of the subgenus discovered so far in the fossil record based on wing venation. Wing venation in *T.* (*D.*) *circumcincta* and *T.* (*D.*) *gorskii* differ significantly and these differences cannot be the result of sexual dimorphism. The most significant are the differences in length of veins Sc, Rs, position of veins sc-r, r-r (R_2_) or the length of the basal section of R_5_. For example the tip of Sc *T.* (*D.*) *gorskii* sp. nov. is short, positioned before fork of Rs while in *T.* (*D.*) *circumcincta* tip of Sc is positioned beyond fork of Rs, crossvein sc-r in *T.* (*D.*) *gorskii* sp. nov. is situated one of its length from edge of wing, in *T.* (*D.*) *baltica* sp. nov. this vein occur in two of its length from edge of wing. In *T.* (*D.*) *gorskii* sp. nov. and *T.* (*D.*) *baltica* sp. nov. Rs arrange more than half of Sc, while in *T.* (*D.*) *circumcintca* Rs is rather short, not longer than half of Sc. The r-r (R_2_) position is also noteworthy, in *T.* (*D.*) *gorskii* sp. nov. r-r (R_2_) is situated beyond fork of Rs while in *T.* (*D.*) *baltica* sp. nov. and *T.* (*D.*) *circumcincta* r-r (R_2_) is situated at the same level as fork of Rs (please, see the comparison section). Even if the structures of the hypopygium of *T.* (*D.*) *circumcincta* and *T.* (*D.*) *gorskii* sp. nov. are similar, the differences in the wing venation definitively indicate that they are different species and can be differentiated and on this basis they can also be distinguished from future new fossil *Dasymolophilus* findings. Although, a characteristic feature of the hypopygium of *T.* (*D.*) *gorskii* sp. nov. is that the gonostylus at midlength is expanded, very elongate, while in *T.* (*D.*) *circumcincta* the gonostylus in midlength is not so widened.

## Data Availability

All data generated or analyzed during this study are included in this published article.
